# Behavioral Monitoring in Transient Ischemic Attack and Stroke Patients: Exploratory Micro- and Macrostructural Imaging Insights for Identifying Post-Stroke Depression with Accelerometers in UK Biobank

**DOI:** 10.3390/s25030963

**Published:** 2025-02-05

**Authors:** Stephanie J. Zawada, Ali Ganjizadeh, Bart M. Demaerschalk, Bradley J. Erickson

**Affiliations:** 1 Mayo Clinic College of Medicine and Science, Scottsdale, AZ 85054, USA; 2 Mayo Clinic Artificial Intelligence Laboratory, Rochester, MN 55905, USA; ganjizadeh.ali@mayo.edu (A.G.); bje@mayo.edu (B.J.E.); 3 Mayo Clinic Department of Neurology, Division of Cerebrovascular Diseases, Phoenix, AZ 85054, USA; demaerschalk.bart@mayo.edu

**Keywords:** stroke, retinal artery occlusion, transient ischemic attack, depression, remote monitoring, sleep

## Abstract

To examine the association between post-stroke depression (PSD) and macrostructural and microstructural brain measures, and to explore whether changes in accelerometer-measured physical activity (PA) are associated with PSD, we conducted an exploratory study in UK Biobank with dementia-free participants diagnosed with at least one prior stroke. Eligible participants (*n* = 1186) completed an MRI scan. Depression was classified based on positive depression screening scores (PHQ-2 ≥ 3). Multivariate linear regression models assessed the relationships between depression and structural and diffusion measures generated from brain MRI scans. Logistic regression models were used to examine the relationship between accelerometer-measured daily PA and future depression (*n* = 367). Depression was positively associated with total white matter hyperintensities (WMHs) volume (standardized β [95% CI]—0.1339 [0.012, 0.256]; FDR-adjusted *p*-value—0.039), periventricular WMHs volume (standardized β [95% CI]—0.1351 [0.020, 0.250]; FDR-adjusted *p*-value—0.027), and reduced MD for commissural fibers (standardized β [95% CI]—−0.139 [−0.255, −0.024]; adjusted *p*-value—0.045). The odds of depression decreased by 0.3% for each daily minute spent in objectively measured light PA, while each minute spent in sleep from midnight to 6:00 AM was associated with a 0.9% decrease in the odds of depression. This early-stage analysis using a population cohort offers a scientific rationale for researchers using multimodal data sources to investigate the heterogenous nature of PSD and, potentially, identify stroke patients at risk of poor outcomes.

## 1. Introduction

Stroke-induced damage to the brain and cerebrovascular system is a well-established risk factor for neurodegenerative conditions, such as long-term disability and future dementia [1]. One modifiable symptom linked to post-stroke cognitive impairment and dementia pathogenesis is depression, with depressed patients being 82% more likely to develop dementia than those without depression [2,3]. Though common in the months and years after stroke, affecting roughly 30% of survivors, post-stroke depression (PSD) remains underdiagnosed and undertreated [3]. Despite the successful validation of numerous instruments to assess depression, including stroke-specific surveys like the Post-Stroke Depression Symptom Inventory (PSDS), the clinical implementation of such tools to screen for PSD is not routine [4,5,6]. Although a growing body of research suggests that PSD screening is a best practice, no consensus has been made regarding the optimal instrument for PSD measurement [5]. The widespread, reliable, and routine detection of PSD remains elusive, limiting the identification of survivors with a modifiable risk factor for poor outcomes.

The heterogenous nature of PSD is one reason for the lack of consensus on tool selection. The classification of depressive symptoms in older adults is also unique, and they sometimes fail to meet DSM criteria [6]. The widespread adoption of minimally invasive remote monitoring tools, such as accelerometers and other wearables, has opened the door to translating objectively measured patterns of behavior—including those linked with depression, like reduced physical activity (PA), increased sedentary behavior (SB), and abnormal sleep patterns—into clinical practice, with the potential to reliably detect patients with PSD [7].

Although research blending active data, like symptom survey sampling, and passive data, such as accelerometer monitoring, after stroke is on the rise, no clear guidelines currently exist [8,9]. In a small-population cohort study of older adult patients (*n* = 76) with minor ischemic stroke, Ashizawa et al. found that depression, as quantified by the Geriatric Depression Scale-15 (GDS-15), was linked with increased SB and decreased light PA, both previously found to be linked with incident dementia [7,10,11]. In a recent study of older adults, including both local (*n* = 63) and national patients (*n* = 280), Stahl et al. found that stroke survivors with high levels of intra-daily variability in their rest–activity rhythms (RARs) were more likely to exhibit depression symptoms captured by the Personal Health Questionairre-9 (PHQ-9), a pattern also established in incident dementia patients [12,13].

Considering prior MRI studies that identified an association between PSD and white matter hyperintensities (WMHs) and white matter microstructural damage, it is plausible that PSD-linked changes in daily behavior and PA may be observable via remote monitoring [14,15,16,17,18,19]. To date, no study has explored relationships between PSD and both (1) brain MRI image-derived phenotypes (IDPs) and (2) accelerometer-measured PA. Additionally, since the release of ICD-11 in 2019, retinal artery occlusion (RAO) has been classified as a type of acute ischemic stroke, highlighting the need for updated post-stroke depression research that includes RAO patients [20,21].

To address these gaps, we conducted a two-part multimodal analysis on a population cohort of post-stroke participants in the UK Biobank (UKBB), the largest lifestyle and mood survey database developed to date. In part one, multivariable linear regression models evaluating associations between macrostructural and microstructural brain MRI IDPs and depression (positive depression screening status) were applied to inform the development of accelerometer-measured daily PA hypotheses. After generating hypotheses from the results of cross-sectional analyses during the imaging assessment, the sample of participants who completed the accelerometer sub-study prior to imaging assessment were filtered for additional analysis in part two. Here, logistic regressions were used to assess hypotheses from part one, evaluating associations between daily behavior and future depression. Through this exploratory study, we aimed to better understand how PA measurement via accelerometer sensors may capture patients with PSD before a depression screening assessment.

## 2. Materials and Methods

### 2.1. Study Population

From 2006 to 2010, over 500,000 middle-aged participants residing in the United Kingdom enrolled in UKBB, an ongoing prospective population cohort study [22]. Informed consent for all UKBB participants was conducted under ethics approval by North West Multi-Centre Research Ethics Committee (reference 21/NW/0157). This research was conducted using the UKBB Resource under application 91159.

**Figure 1:**
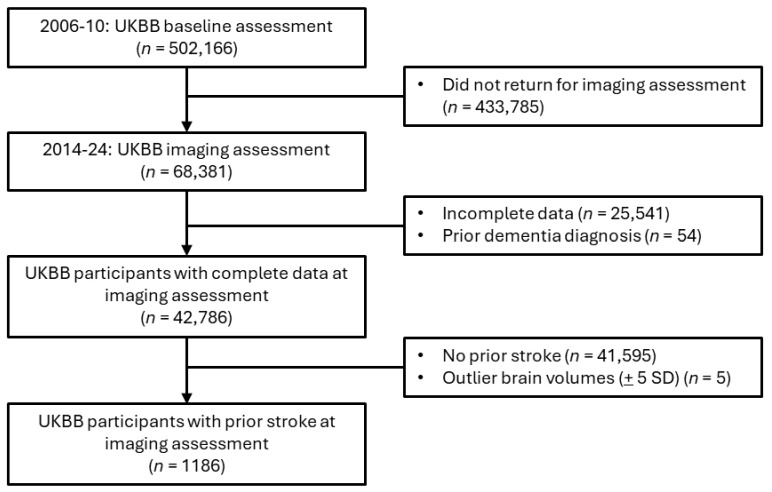
Cohort filtering for imaging statistical and sensitivity analyses.

**Figure 2:**
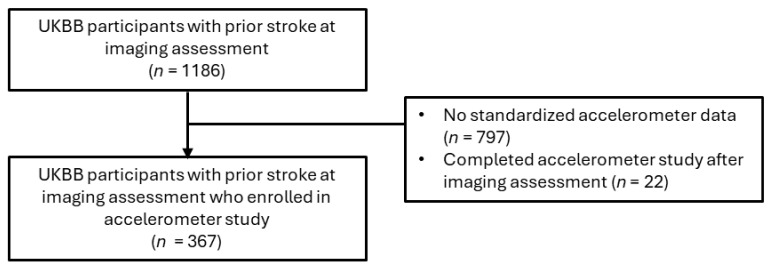
Cohort filtering for accelerometer statistical and sensitivity analyses.

For the primary analysis assessing associations between brain MRI IDPs and depression after stroke, participants with a prior dementia diagnosis were excluded, given that patients with dementia frequently exhibit negative effects or mood changes (Figure 1). The included participants were those who had suffered at least one stroke (*n* = 1186), including ischemic stroke, RAO, hemorrhagic stroke, cryptogenic stroke, or transient ischemic attack (TIA). A subsample of the eligible imaging study participants (*n* = 367) completed the accelerometer study before the PHQ-2 screening conducted during the imaging assessment (Figure 2).

### 2.2. Data Collection

Demographic (age, sex, ethnicity, education level), lifestyle (weekly leisure/social activities, smoking status, sleeplessness/insomnia, snoring, nightly sleep hours), prescription (beta blocker use), and medical/laboratory (systolic blood pressure, body mass index) data were collected at the time of imaging assessment (i.e., from 2014 to 2024). The following diagnoses were aggregated from linked hospital and general practitioner records, as well as self-report surveys and interviews at the imaging assessment: stroke, depression, dementia, hypertension, hyperlipidemia, non-stroke cerebrovascular disease (CeVD), and stroke sequelae (Supplementary Table S1). Hypertension was also defined using the average of two systolic blood pressure readings taken at the imaging visit (≥140 mm Hg). Time from initial stroke to MRI scan was calculated by subtracting the date of a participant’s first stroke from the date of imaging assessment.

Dichotomized variables included ethnicity (white vs. non-white), education level (college/university degree vs. no college/university degree), smoking status (current/former smoker vs. no smoking history), snoring (yes/no), weekly leisure/social activities (yes/no), full ischemic or hemorrhagic stroke (yes/no), multiple strokes (yes/no), and diagnostic status (yes/no) for depression, hypertension, hyperlipidemia, and non-stroke CeVD or stroke sequelae.

As beta-blocker medications may influence the development of depression, their prescribed use was recorded as a dichotomous variable. Severity of sleeplessness/insomnia was stratified (usually vs. sometimes vs. never/rarely). Weekly leisure/social activities included sports club or gym, pub or social club, religious group, adult education class, or other group activity.

Though demographic, lifestyle, and medical/laboratory data were not sampled at the time of the accelerometer study, conducted from 2013 to 2015, age and diagnostic statuses were updated. The median time between the accelerometer sub-study and the imaging assessment was 3.3 years (range, 0–8.2 years; IQR = 1.8–4.3).

### 2.3. Depression Variables

Recommended for PSD screening by the American Stroke Association due to its ease-of-use and accuracy, the validated two-item PHQ-2 survey was used to measure depression at the imaging assessment, capturing previously undiagnosed depression or early signs of depression [23,24,25].

A score of 3 or more was used for categorizing patients with depression (positive depression screen), having been previously established as the PHQ-2’s optimal cutoff score [26,27]. Regarding the PHQ-2 survey, participants who chose not to respond were dropped from the dataset. When participants reported “Do not know” for either survey question, their response was recoded from ‘−1’ to ‘0’.

### 2.4. Brain MRI Measures

Between 2014 and 2024, brain MRI scans were acquired using a Siemens Skyra 3 T scanner (Siemens Medical Solutions USA, Inc., Malvern, PA, USA) with 32-channel receive head coil. Detailed descriptions of the UKBB image acquisition protocols are available online [28,29,30]. Both T1-weighted and T2-weighted FLAIR structural imaging were performed to generate brain structure volumes. Imaging-derived phenotypes (IDPs) were generated from raw imaging data by UKBB researchers using Freesurfer (v. 6.0) and FMRIB Software Library (v. 5.0.10) pipelines. The macrostructural IDPs in this study are total brain, grey matter, white matter, WMHs, deep WMHs, periventricular WMHs, peripheral cortical grey matter, and subcortical structures (accumbens, amygdala, caudate, hippocampus, pallidum, putamen, thalamus). To capture effect sizes as standardized values, all volumes were rescaled into 0 mean and unitary standard deviation.

To approximate white matter microstructural integrity, diffusion tensor imaging was used [28,29,30]. Additional IDPs included fractional anisotropy (FA) and mean diffusivity (MD) for summarized measures of global fibers, association fibers, commissural fibers, and projection fibers. This approach was consistent with those of prior UKBB studies, using 27 white matter tracts [30]. Individual white matter fibers were filtered into one of three white matter tracts—commissural fibers, projection fibers, and association fibers [30] (Table 1).

The means of both hemispheres were calculated for bilateral subcortical structures. Log transformation of WMHs was performed to adjust for a positively skewed distribution. Volumes beyond ±5 standard deviations from the mean were excluded [31]. Confounders also generated by UKBB were the head-scanner position and the head-size scaling factor. Within the subsample of participants who completed the imaging study protocol (*n* = 42,786), only 1186 participants had previously suffered a stroke (Figure 1).

### 2.5. Accelerometer Data

Among imaging participants who enrolled in the 7-day accelerometer sub-study, 367 participants were eligible based on a prior stroke diagnosis. Participants were mailed the Axivity AX3 wristwatch (Axivity Ltd., Newcastle, UK) with 100 Hz logging to wear on their dominant arm. A pamphlet instructing participants to wear the accelerometer continuously for one week was also included. During 2013–2015, participants conducted themselves normally for a 1-week observation period, allowing the watch’s accelerometer sensors to capture 3-dimensional data (*x*-*y*-*z* axes) with a sensitivity range of ±8 g. Using prior UKBB accelerometer quality control, physical activity filtering, and imputation protocols, sensor data yielded time spent in 4 PA categories—sleep, sedentary behavior (SB), light (L)PA, and moderate-to-vigorous (MV)PA (https://biobankaccanalysis.readthedocs.io/ accessed on 1 October 2024).

To calibrate the accelerometer data for analysis, we applied a similar protocol to the one proposed by Madjedi et al. [32]. First, only participants who wore the accelerometer for 3 or more days were included. Next, only those participants for whom raw accelerometer data was recorded during every hour, for the duration of their participation, were included. Then, participants whose accelerometers recorded outlier acceleration (>100 mg) were excluded. Lastly, participants with accelerometer data compromised by more than 1% clips (readings with acceleration exceeding a magnitude of 8 g) were excluded.

### 2.6. Statistical Analysis

Data wrangling and analyses were performed in Python 3.12 using statistical packages (*SciPy*, *NumPy*, *Pandas*, and *Statsmodels*). The *fancyimpute* package was used to implement multiple chain imputation for missing data for the following covariables: BMI (2.28% incomplete), systolic blood pressure (22.2% incomplete), diastolic blood pressure (22.2% incomplete), total intracranial volume (2.12% incomplete), fractional anisotropy (2.03% incomplete), mean diffusivity (2.03% incomplete), periventricular WMHs (0.08% incomplete), deep WMHs (0.08% incomplete), brainstem volume (3.12%), cerebrospinal fluid (3.12%), thalamus volume (3.12% incomplete), caudate volume (3.12% incomplete), putamen volume (3.12% incomplete), pallidum volume (3.12% incomplete), hippocampus volume (3.12% incomplete), amygdala volume (3.12% incomplete), and accumbens volume (3.12% incomplete).

Summarized characteristics obtained upon imaging assessment between participants with and without positive depression screen scores were examined using Mann-Whitney and chi-square tests for continuous and categorical variables, respectively (Table 2). Considering covariance across white matter microstructural measures, confirmatory factor analysis (CFA) was applied to generate a summary latent measure for the FA and MD of each main nerve tract (association fibers, commissural fibers, and projection fibers) and a summary white matter variable (global fibers).

Adjusting for age, sex, BMI, stroke severity, time from initial stroke to MRI scan, head-scanner position (including *x*-*y*-*z* brain position), intracranial volume, and head-size scaling factor (ratio of volumetric scaling from T1 head image to standard space), multiple linear regression models estimated beta coefficients (standardized) with 95% confidence intervals (CIs) for associations between PHQ-2-measured depression (yes/no) and (1) macrostructural (MRI) brain volumes and (2) microstructural integrity (dMRI weighted means). False discovery rate correction (Benjamini and Hochberg) was applied to linear regression outputs with corrected *p*-values <0.05 classified as significant. The stroke severity variable recorded the most severe stroke event in a participant’s history (cerebral infarction or hemorrhagic stroke as the most severe and TIA as the least severe).

When significant associations were identified, hypotheses related to observable behavioral changes were developed and investigated using logistic regression to assess whether in situ daily or hourly accelerometer data could predict PHQ-2-measured depression status. Logistic regression models were adjusted for age, sex, time from initial stroke to accelerometer study, and season of accelerometer wear. Odds ratios (ORs) were reported with 95% CIs, assessing the influence of PA behaviors (MVPA, LPA, SB, and sleep) on future depression status.

### 2.7. Sensitivity Analysis

Regarding WMHs volume, another sensitivity analysis was performed to investigate deep WMHs and periventricular WMHs, considered exclusive of one another. In a second sensitivity analysis, the microstructural and macrostructural imaging analyses were conducted without participants who had a prior depression diagnosis. An accelerometer sensitivity analysis was performed, assessing hourly sleep patterns between the hours most persons sleep—12:00–5:59 AM (or 0:00–5:59 h) [33].

## 3. Results

### 3.1. Participant Characteristics

Compared to participants who did not screen positive for depression, a higher proportion of depressed participants (*n* = 274) were younger, a current or former smoker, non-white, and experienced frequent insomnia (Table 2). A higher proportion of depressed participants also had a prior depression diagnosis and a prior full stroke (ischemic or hemorrhagic). In the depressed cohort, participants had a more recent initial stroke, a higher BMI, and reduced sleep hours, on average. A lower proportion of depressed participants engaged in one or more social activity per week. The average age of participants in both cohorts was greater than 65. Imaging assessment characteristics, comparing cohorts, were obtained by conducting Mann–Whitney and χ^2^ tests for continuous and categorical covariables (Table 2).

No statistically significant differences between depressed and non-depressed cohorts were observed for sex, college/university education, hypertension diagnosis, hyperlipidemia diagnosis, prior non-stroke CeVD or stroke sequelae diagnosis, snoring or beta-blocker use (Table 2). All participants had more than one prior stroke diagnosis.

### 3.2. Depression and Structural Brain Differences

Compared to those who screened negative for depression, depression was positively associated with WMHs volume (total) (standardized β [95% CI]—0.1339 [0.012, 0.256]; FDR-adjusted *p*-value—0.039) (Table 3).

Adjusting for relevant covariables, the relationships between depression and FA (global and regional fibers) were not statistically significant (Table 4). Depression was also associated with reduced MD for commissural fibers (standardized β [95% CI]—0.139 [−0.255, −0.024]; adjusted *p*-value—0.045) (Table 5).

### 3.3. In Situ Accelerometer-Measured Daily Activity and Depression

For each minute spent in light physical activity per day, the odds of screening positive for depression at the imaging assessment decreased by 0.3% (Table 6). No statistically significant relationships were identified between depression status and daily MVPA, sedentary, or sleep duration.

Regarding 24-h sleep patterns, each minute spent in sleep from midnight to 6:00 AM was associated with a 0.9% decrease in the odds of screening positive for depression (Table 7).

### 3.4. Sensitivity Analyses

In the first sensitivity analysis, we expanded on the multivariate linear regression models to investigate sub-types of WMHs, as follows: deep WMHs and periventricular WMHs. Depression was only associated with periventricular WMHs volume (standardized β [95% CI]—0.1351 [0.020, 0.250]; FDR-adjusted *p*-value—0.027) (Supplementary Table S5).

In another sensitivity analysis, we completed the imaging analyses without participants who had a prior depression diagnosis (*n* = 1022) (Supplementary Table S6). WMHs (standardized β [95% CI]—0.146 [0.008, 0.284]; adjusted *p*-value—0.047) and periventricular WMHs (0.146 [0.015, 0.277]; adjusted *p*-value = 0.035) were the only macrostructural volumes with a significant association with depression. The association between depression and reduced MD for commissural fibers also persisted (−0.165 [−0.294, −0.036]; adjusted *p*-value = 0.012).

In an accelerometer sensitivity analysis, we further probed sleep patterns between 0:00–5:59 h (Supplementary Table S7). No associations were found for depression and 0:00–0:59 h, 1:00–1:59 h or 5:00–5:59 h. For 2:00–2:59 h (OR = 0.972 [0.949–0.995]), 3:00–3:59 h (OR = 0.952 [0.917–0.988]), and 4:00–4:59 h (OR = 0.947 [0.904–0.991]), sleep (min) was slightly inversely associated with depression.

## 4. Discussion

Using a population cohort from the UKBB imaging study of post-stroke patients, we found that a positive depression screening score (measured by PHQ-2) was linked to overall deteriorated brain health, and that post-stroke depression may be observable before screening with an accelerometer. Depression was associated with macrostructural markers of neurodegeneration, including total WMHs as well as periventricular WMHs, and microstructural markers, namely, decreased MD in commissural fibers. Based on these findings, we hypothesized that less time in daily LPA and MVPA would be associated with depression, and that irregular sleep patterns would be observable. Though no association was found for MVPA and depression, we confirmed that LPA and depression status were inversely associated, and that decreased sleep during late night/early morning (midnight–5:59 AM) was associated with depression.

Stroke patients commonly experience poor sleep, a risk factor for long-term disability and poor outcomes. In some cases, poor sleep may be a symptom of PSD, or vice versa. To date, most of the literature published on post-stroke sleep focuses on single-site, short-term studies assessing specific sleep disorders, like sleep apnea and insomnia [34,35,36,37]. Our findings expand on these studies by examining PSD symptoms observable roughly 7 years after an initial stroke event. Considering that all participants in our study suffered more than 1 stroke event prior to the imaging visit, our study presents a long-term lifespan view of PSD, which has previously only rarely been examined [38].

Additionally, some symptoms of stroke or of depression may overlap and be indistinguishable from each other. One such symptom, poor sleep quality, has been linked to the development of persistent PSD. Fan et al. found that differences in depression and anxiety stratified by sleep quality were not statistically significant at baseline (2 weeks after hospitalization); however, at the end of 3 months, those with poor sleep had a higher risk of PSD and anxiety. Our results extend these findings, suggesting that PSD may persist for years after a stroke [34].

As some symptoms of stroke and depression overlap at different times in the rehabilitation phase, it is often challenging to distinguish between temporary and persistent cases. Moreover, symptoms may go unnoticed by patients with cognitive impairment or dementia, especially in older patients. Considering that only 20% of PSD cases are identified by clinicians who are not psychiatrists, the need for strategies to identify PSD at scale is urgent [39,40].

To address the underdiagnosis of PSD, our results provide a scientific rationale for the targeted monitoring of sleep and PA to identify patients who would benefit from depression screening. Firstly, some research suggests that disturbed nighttime sleep patterns, such as those identified in our study and by others, may be a distinct phenotype of PSD not found in depressed patients without a stroke history [41]. Liu et al. found that less than 6 h of sleep per night was associated with depression after ischemic stroke [42]. Our work expands on this finding, highlighting the role of reduced late night/early morning sleep in the development of depressive symptoms. These results underscore the potential for sleep treatments to treat PSD, and thereby improve stroke outcomes.

Next, stroke is implicated in the dysregulation of the autonomic nervous system (ANS), which plays a role in sleep initiation and maintenance. As such, aberrations in ANS activity may lead to behavioral changes (which can be captured by accelerometers) that induce depressive symptoms or increase the risk of other CeVDs [43]. The results of the accelerometer sub-study (namely, (1) the association between late night/early morning sleep and depression and (2) no significant association between total daily sleep and depression) may indirectly capture this phenomenon, as they suggest depressed patients may experience sleep disturbances. Objectively measured sleep patterns, such as those captured by accelerometers, may identify sleep disturbances, especially in patients with memory decline, cognitive impairment or emerging dementia, which would otherwise go undetected. Also taking into account the decreased MD in commissural fibers, the participants in our study likely experienced reduced slow wave sleep, also known as deep sleep, a component of memory consolidation and metabolic waste clearing processes [44]. Avvenuti et al. confirmed that the microstructural integrity of the corpus callosum, the main commissural tract, is necessary for sleep slow waves to occur in both brain hemispheres [45]. The reduced MD in commissural fibers and sleep disturbance observed in our study may facilitate the buildup of proteins like amyloid-β and tau proteins, thereby accelerating neurodegeneration processes [46]. Specific to dementia, poor sleep is an established modifiable risk factor. For adults ages 60 and older, Himali et al. found that even a 1% decrease in nightly slow wave sleep increases the risk of dementia by 27% [46]. This work highlights the potential relevance of our findings, which, although only detecting slight aberrations in behavior in the depressed cohort, may offer a scientific rationale for sensor-based research in the post-stroke space.

Alternatively, WMHs may induce sleep problems, which then result in microstructural changes. Suboptimal myelination may lead to decreased microstructural integrity, via which depression may emerge as a symptom [47]. WMHs have been extensively characterized as a risk factor for stroke and cognitive decline, including incident dementia [48,49]. Numerous studies have also identified an association between depression and WMHs, which may influence the effectiveness of depression treatment [50]. A recent meta-analysis found links between WMHs and both cross-sectional and incident depression [51]. Although WMHs are a marker of neurodegeneration, the results of both the Rotterdam and the Framingham Offspring Studies suggest that WMHs may be the result of broader, or system-wide, vascular dysfunction, regardless of stroke or dementia [52,53,54,55]. Our results confirm those of previous studies regarding the link between WMH and depression, as well as periventricular WMHs and PSD [56]. Additionally, Zong et al. found a positive relationship between renal dysfunction and periventricular WMHs severity in stroke/TIA patients [57]. Considering these findings and that the pathogenesis of periventricular WMHs is distinct from that of deep WMHs, WMHs, especially periventricular WMHs, may be the result of complex vascular changes.

Though WMHs may increase with age, research suggests that physical activity may play a role in preventing or slowing their development [58]. Since in our study, WMHs were linked to depression, we hypothesized that reduced MVPA and LPA would both be associated with depression. Surprisingly, we only found a link with LPA, a discovery previously confirmed [59]. One plausible explanation is that post-stroke patients engage in less MVPA than the general population. For instance, in a small prospective study (*n* = 40), Tanaka et al. found that post-stroke patients spend only 1% of their time in MVPA compared to 8% in LPA [60]. In our study, depressed participants were less likely to engage in at least one social activity per week (69.7 vs. 77.9%; *p*-value = 0.007). As such, social activity—including commuting, walking, and interacting with others—may be one way post-stroke participants engage in LPA that contributes to a reduction in PSD risk.

The strengths of this study include the sample size for neuroimaging data; however, the sample size for the accelerometer study is small, limiting the interpretation of the respective findings. To date, no UKBB study assessing post-stroke depression has been published. Another strength of our study design is that, when excluding patients with a prior depression diagnosis, the associations between a positive depression screening score and both micro- and macrostructural measures persisted, suggesting that PSD may emerge even in patients with a prior depression diagnosis. Alternatively, a depression diagnosis before stroke may be a risk factor for PSD [61]. One major limitation of this study is the self-reported data obtained at the imaging assessment for lifestyle factors, considering that self-reported assessments are inherently subject to bias and inaccuracies. At the imaging assessment, no blood biochemistry samples were taken, restricting diagnoses of hyperlipidemia to records and self-reporting and limiting the physical biomarkers available to be included as confounders. The PHQ-2 survey itself is a short assessment of only two questions and, while useful in fast-paced, short-staffed environments, may inadvertently be biased against certain types of PSD. Moreover, standardized stroke severity scores, like NIHSS, were not available in the UKBB. As stroke severity is a known risk factor for PSD, we stratified stroke severity in a tailored way that may be insufficient given its localized and transient nature, considering hemorrhagic stroke or cerebral infarction to be the most severe type of stroke and TIA to be the least severe.

Another limitation is that the imaging data were not collected across multiple time points to facilitate a longitudinal analysis of brain structure and volume in PSD. This unfortunately limited the study design to cross-sectional analyses, which cannot capture the progression of PSD. An additional limitation is the fact that UKBB participants in the imaging and accelerometer studies are known to be younger, and had fewer comorbidities than those participating in the initial baseline assessment (without imaging). As the UKBB study population is primarily White and of European origin, the findings from this study may not generalize well to individuals of other racial or cultural backgrounds [61,62]. Given that the optimal time for sampling depressed mood in post-stroke patients via validated instruments is unknown, the clinical usefulness of assessing PSD years after an initial stroke event is unknown; based on our findings, the use of PHQ-2, as recommended by the literature, appears to be a feasible method of screening for PSD. Additionally, as TIA is frequently underdiagnosed, participants with undiagnosed TIA may have been filtered from our analysis at the start [63].

## 5. Conclusions

Based on the early-stage evidence from our study, the PHQ-2 appears to correlate with directly measured microstructural and macrostructural differences in the brains of patients with PSD and with behavioral differences measured indirectly and in situ via accelerometers. WMHs, particularly periventricular WMHs, were associated with depression, and reduced MD in the commissural fibers was as well. Accelerometer-measured hourly sleep patterns appear to be disrupted in depressed patients, while time spent in daily LPA was inversely associated with depression. The combination of multimodal data sources, including accelerometer monitoring, imaging, and survey screening, may one day generate clinically actionable insights into the heterogenous nature of PSD, thereby identifying more patients at risk for PSD and other poor functional outcomes.

## Figures and Tables

**Table 1:** White matter tract components.

Regional White Matter Tract	Specific White Matter Fibers
Commissural Fibers	Anterior Thalamic Radiation (Left and Right), Superior Thalamic Radiation (Left and Right), Posterior Thalamic Radiation (Left and Right)
Projection Fibers	Acoustic Radiation (Left and Right), Forceps Major, Forceps Minor, Middle Cerebellar Peduncle, Medial Lemniscus (Left and Right), Corticospinal Tract (Left and Right)
Association Fibers	Parahippocampal Cingulum (Left and Right), Cingulate Gyrus of Cingulum (Left and Right), Inferior Fronto-Occipital Fasciculus (Left and Right), Inferior Longitudinal Fasciculus (Left and Right), Superior Longitudinal Fasciculus (Left and Right), Uncinate Fasciculus (Left and Right)

**Table 2:** Participant characteristics at imaging assessment.

	Controls (*n* = 912)	Positive Depression Screen (*n* = 274)	*p*-Value
Age (y)	70 [65, 74]	69 [62, 73]	<0.001
Sex (male)	62.10%	58.00%	0.361
White	98.00%	95.60%	<0.001
BMI (kg/mm^2^)	26.5 [23.9, 29.3]	27.0 [24.3, 29.9]	<0.001
Time from Initial Stroke to MRI Scan (y)	7.79 [3.68, 14]	7.43 [3.66, 13.95]	<0.001
Multiple Strokes	100%	100%	1
Ischemic or Hemorrhagic Stroke	51.60%	58.80%	0.007
College/University Education	45.00%	40.10%	0.181
Weekly Social Activity	77.90%	69.70%	0.007
Smoker Status			0.013
Never	59.10%	51.10%	
Previous	38.60%	44.20%	
Current	2.20%	4.70%	
Sleeplessness			<0.001
Never/Rarely	24.60%	10.60%	
Sometimes	45.30%	42.00%	
Usually	29.80%	47.40%	
Snoring	58.90%	54.00%	0.133
Sleep (h/day)	7 [7,8]	7 [6,8]	<0.001
Diagnosed Depression	9.70%	25.90%	<0.001
Hypertension	88.60%	92.00%	0.139
Hyperlipidemia	61.00%	60.20%	0.880
Non-Stroke Cerebrovascular Disease or Stroke-Related Sequelae	10.90%	13.90%	0.207
Beta-Blocker Use	12.10%	13.90%	0.490
Antidepressant Use	4.00%	13.60%	<0.001

Data are presented as median [P25, P75] or proportion (%).

**Table 3:** Multivariate linear regression for brain volumes.

	Positive Depression Screen			
	Yes		No	
Brain Regions	β (95% CI)	*p*-Value	β (95% CI)	*p*-Value
White Matter Hyperintensities	0.1319 (0.010, 0.254)	0.042	Reference	
Total Brain	0.0259 (−0.085, 0.137)	0.746	Reference	
Peripheral Cortical Grey Matter	−0.0301 (−0.131, 0.070)	0.526	Reference	
Total Grey Matter	−0.0312 (−0.130, 0.067)	0.495	Reference	
Total White Matter	0.1024 (−0.027, 0.232)	0.237	Reference	
Brainstem Volume	0.0736 (−0.028, 0.175)	0.538	Reference	
Cerebrospinal Fluid	−0.0518 (−0.166, 0.062)	0.423	Reference	
Thalamus (L)	−0.0402 (−0.142, 0.061)	0.466	Reference	
Thalamus (R)	−0.0369 (−0.138, 0.065)	0.562	Reference	
Caudate (L)	0.0038 (−0.103, 0.111)	0.779	Reference	
Caudate (R)	0.0544 (−0.054, 0.163)	0.505	Reference	
Putamen (L)	0.0863 (−0.027, 0.200)	0.329	Reference	
Putamen (R)	0.0859 (−0.029, 0.200)	0.199	Reference	
Pallidum (L)	0.0585 (−0.055, 0.172)	0.439	Reference	
Pallidum (R)	0.1023 (−0.009, 0.213)	0.071	Reference	
Hippocampus (L)	−0.0478 (−0.157, 0.062)	0.624	Reference	
Hippocampus (R)	−0.0583 (−0.169, 0.053)	0.814	Reference	
Amygdala (L)	−0.0897 (−0.199, 0.020)	0.349	Reference	
Amygdala (R)	−0.0697 (−0.179, 0.039)	0.528	Reference	
Accumbens (L)	−0.0067 (−0.119, 0.105)	0.807	Reference	
Accumbens (R)	−0.0926 (−0.207, 0.022)	0.21	Reference	

Adjusted for age, sex, BMI, stroke severity, time from initial stroke to MRI scan, head-scanner position, intracranial volume, and head-size scaling factor. R and L denote right and left, respectively.

**Table 4:** Multivariate linear regression for fractional anisotropy.

Positive Depression Screen	Fractional Anisotropy (FA)							
	Global FA		Association Fibers		Commissural Fibers		Projection Fibers	
	β (95% CI)	*p*-Value	β (95% CI)	*p*-Value	β (95% CI)	*p*-Value	β (95% CI)	*p*-Value
Yes	0.0063 (−0.121, 0.134)	0.923	0.0303 (−0.095, 0.156)	0.762	0.0474 (−0.078, 0.172)	0.628	−0.0117 (−0.128, 0.104)	0.881
No	Reference		Reference		Reference		Reference	

Adjusted for age, sex, BMI, stroke severity, time from initial stroke to MRI scan, head-scanner position, intracranial volume, and head-size scaling factor.

**Table 5:** Multivariate linear regression for mean diffusivity.

Positive Depression Screen	Mean Diffusivity (MD)							
	Global MD		Association Fibers		Commissural Fibers		Projection Fibers	
	β (95% CI)	*p*-Value	β (95% CI)	*p*-Value	β (95% CI)	*p*-Value	β (95% CI)	*p*-Value
Yes	0.0713 (−0.049, 0.192)	0.285	0.0336 (−0.086, 0.154)	0.647	−0.1393 (−0.255, −0.024)	0.045	−0.0246 (−0.142, 0.093)	0.681
No	Reference		Reference		Reference		Reference	

Adjusted for age, sex, BMI, stroke severity, time from initial stroke to MRI scan, head-scanner position, intracranial volume, and head-size scaling factor.

**Table 6:** Summary measures for daily PA and odds ratios for daily PA and depression.

			Positive Depression Screen			
			Yes		No	
Accelerometer-Measured Behavior	Positive Depression Screen (min/day)	Controls (min/day)	Odds Ratio	*p*-Value	Odds Ratio	*p*-Value
MVPA	39.5 (30.0)	42.3 (36.9)	1.002 (0.999–1.004)	0.180	Reference	
SB	583.3 (112.6)	568.3 (116.6)	1.001 (0.999, 1.004)	0.281	Reference	
LPA	259.7 (100.9)	288.7 (107.4)	0.997 (0.994–0.999)	0.014	Reference	
Sleep	557.5 (122.6)	540.7 (100.7)	0.999 (0.999, 1.007)	0.848	Reference	

Min/day data are presented as mean (standard deviation). MVPA = Moderate-to-Vigorous Physical Activity; SB = Sedentary Behavior; LPA = Light Physical Activity. Odds ratios are adjusted for age, sex, time from initial stroke to accelerometer study, and season of wear.

**Table 7:** Time epoch measures for daily sleep and odds ratios for time-epoch sleep and depression.

	Positive Depression Screen			
	Yes		No	
Time of Day for Sleep (h)	Odds Ratio	*p*-Value	Odds Ratio	*p*-Value
0:00–5:59	0.991 (0.984–0.997)	0.005	Reference	
6:00–11:59	1.005 (1.000–1.009	0.074	Reference	
12:00–17:59	1.004 (0.996–1.012)	0.331	Reference	
18:00–23:59	1.002 (0.997–1.007)	0.454	Reference	

Odds ratios are adjusted for age, sex, time from initial stroke to accelerometer study, and season of wear; 0:00 h indicates 12:00 AM.

## Data Availability

All anonymized data collected are available as open data via an application to UK Biobank data repository.

## Supplementary Materials

The following supporting information can be downloaded at: https://www.mdpi.com/article/10.3390/s25030963/s1, Table S1: Diagnosis definitions; Table S2: Antidepressant names and Rx codes in UKBB; Table S3: Beta-blocker names and Rx codes in UKBB; Table S4: PHQ-2 survey questions; Table S5: Multivariate linear regression for WMHs volumes; Table S6: Multivariate linear regression for brain and WMHs volumes (prior depression diagnosis excluded) (n = 1022); Table S7: Odds ratios for hourly sleep and depression.

## Abbreviations

The following abbreviations are used in this manuscript:

TIA	Transient ischemic attack
PSD	Post-stroke depression
PHQ-2	Patient Health Questionnaire-2
PA	Physical activity
MVPA	Moderate-to-vigorous physical activity
LPA	Light physical activity
SB	Sedentary behavior (PA)
WMHs	White matter hyperintensities
BMI	Body mass index
UKBB	UK Biobank
FA	Fractional anisotropy
MD	Mean diffusivity
IDP	Image-derived phenotype
RAO	Retinal artery occlusion
CeVD	Cerebrovascular disease
FDR	False discovery rate
OR	Odds ratio
ANS	Autonomic nervous system
