# Supplementary material for: Behavioral Monitoring in Transient Ischemic Attack and Stroke Patients: Exploratory Micro- and Macrostructural Imaging Insights for Identifying Post-Stroke Depression with Accelerometers in UK Biobank
Source: Sensors (Basel). 2025 Feb 5;25(3):963. doi: 10.3390/s25030963 (PMC11820421; doi:10.3390/s25030963)
Supplement: Supplementary file 1 [file sensors-25-00963-s001.zip › sensors-3415820-supplementary.pdf]

Supplementary Table S1. Diagnosis Definitions.

**Diagnosis**	***ICD-10* code**	**Self-reported medical history**	**Imaging Assessment Sample**
Hyperlipidemia	E7	1473	
Dementia (all types)	F00, F01, F02, F03	None	
Depression	F32, F33, F34, F38, F39	1286	
TIA	G45	1082	
Vascular syndromes of CeVD	G46	None	
Retinal artery occlusion	H34	1282	
Hypertension	I10, I15	1065, 1072	Systolic Blood Pressure > 140
Subarachnoid hemorrhage	I60	1086	
Intracerebral hemorrhage	I61	1491	
Other hemorrhage	I62	1491, 1083	
Cerebral infarction	I63	1583	
Unspecified stroke	I64	1081	
Precerebral arterial occlusion/stenosis	I65	None	
Cerebral arterial occlusion/stenosis	I66	None	
Other CeVD	I67	None	
Other cerebrovascular disorders	I68	None	
CeVD sequelae	I69	None	

Supplementary Table S2. Antidepressant Names and Rx Codes in UKBB.

**Antidepressant**	**Rx Code in UKBB**
amitriptyline	1140879616
citalopram	1140921600
fluoxetine	1140879540
sertraline	1140867878
venlafaxine	1140916282
dosulepin	1140909806
paroxetine	1140867888
mirtazapine	1141152732
escitalopram	1141180212
trazodone	1140879634
prozac	1140867876
seroxat	1140882236

cipralex	1141190158
duloxetine	1141200564
lofepramine	1140867726
clomipramine	1140879620
nortriptyline	1140867818
imipramine	1140879630
dothiepin	1140879628
cipramil	1141151946
amitriptyline	1140867948
prothiaden	1140867624
trimipramine	1140867756
lustral	1140867884
reboxetine	1141151978
zispin	1141152736
cymbalta	1141201834
anafranil	1140867690
doxepin	1140867640
moclobemide	1140867920
phenelzine	1140867850
fluvoxamine	1140879544
yentreve	1141200570
triptafen	1140867934
surmontil	1140867758
tranylcypromine	1140867914
allegron	1140867820
edronax	1141151982
molipaxin	1140882244
mianserin	1140879556
nardil	1140867852
faverin	1140867860
nefazodone	1140917460
amitriptyline+chlordiazepoxide	1140867938
isocarboxazid	1140867856
manerix	1140867922
maoi	1140910820
sinequan	1140882312
tranylcypromine+trifluoperazine	1140867944
ludiomil	1140867784
norval	1140867812
tryptizol	1140867668

Supplementary Table S3. Beta-Blocker Names and Rx Codes in UKBB.

**Beta-blocker**	**Rx Code in UKBB**
Atenolol	1140866738
bisoprolol	1140879760
metoprolol	1140879818

carvedilol	1140909368
propranolol	1140879842
inderal 10mg tablet	1140866804
apsolol 10mg tablet	1140866764
propanix 10mg tablet	1140866766
sotalol	1140879854
nebivolol	1141164276
dorzolamide+timolol	1141169516
atenolol+bendroflumethiazide	1141194810
latanoprost+timolol	1141184722
betaxolol	1140879758
atenolol+bendrofluazide	1141146126
nadolol	1140860192
prindolol	1140910614
Timolol	1140875840
ethambutolol	1140884298
pindolol	1140860292
atenolol+chlortalidone	1141180778
atenolol+nifedipine 50mg/20mg m/r capsule	1140860426
atenolol+chlorthalidone	1141146124
atenolol+co to amilozide	1141146128
nadolol+bendroflumethiazide 40mg/5mg tablet	1141194804
timolol maleate+bendroflumethiazide 10mg/2.5mg tablet	1141194808
bisoprolol fumarate+hydrochlorothiazide 10mg/6.25mg tablet	1140864950
celiprolol	1140879762
labetalol	1140879824
oxprenolol	1140879830
acebutolol	1140866724
propranolol hydrochloride+bendrofluazide 80mg/2.5mg capsule	1140860418
sotalol hydrochloride+hydrochlorothiazide 80mg/12.5mg tablet	1140860332
metoprolol tartrate+hydrochlorothiazide 100mg/12.5mg tablet	1140860404
beta to blocker	1140916342
tenormin 25 tablet	1140866756
bedranol 10mg tablet	1140851556
levobunolol	1140879826
sotalol hydrochloride+hydrochlorothiazide 80mg/12.5mg tablet	1140860332
propranolol hydrochloride+bendrofluazide 80mg/2.5mg capsule	1140860418
carteolol	1140879822
metoprolol tartrate+chlorthalidone 100mg/12.5mg tablet	1140860308
half beta to prograne 80mg m/r capsule	1140866802
beta to prograne 160mg m/r capsule	1140866782
timolol maleate+co to amilozide 10mg/2.5mg/25mg tablet	1140860336
cardinol 10mg tablet	1140866712

Supplementary Table S4. PHQ-2 Survey Questions.

		Score			
**Question No.**	**Over the last 2 weeks, how often have you been bothered by any of the following problems?**	**Not at all**	**Several Days**	**More than half the days**	**Nearly Every Day**
1	Little interest or pleasure in doing things	0	1	2	3
2	Feeling down, depressed, or hopeless	0	1	2	3

Supplementary Table S5. Multivariate Linear Regression for WMHs Volumes.

	**Depression**			
	**Yes**		**No**	
**Brain Regions**	**β (95 % CI)**	**p-value**	**β (95 % CI)**	**p-value**
Deep White Matter Hyperintensities	0.0872 (-0.097, 0.271)	0.392	Reference	
Periventricular White Matter Hyperintensities	0.1351 (0.020, 0.250)	0.027	Reference	

Adjusted for age, sex, BMI, stroke severity, time from initial stroke to MRI scan, head-scanner position, intracranial volume, and head-size scaling factor

Supplementary Table S6. Multivariate Linear Regression for Brain and WMHs Volumes (Prior Depression Diagnosis Excluded) (n = 1022).

	**Depression**			
	**Yes**		**No**	
**Brain Regions**	**β (95 % CI)**	**p-value**	**β (95 % CI)**	**p-value**
White Matter Hyperintensities	0.1501 (0.012, 0.288)	0.034	Reference	
Total Brain	-0.0244 (-0.151, 0.103)	0.220	Reference	
Peripheral Cortical Grey Matter	-0.0725 (-0.188, 0.043)	0.526	Reference	
Total Grey Matter	-0.0770 (-0.191, 0.037)	0.185	Reference	
Total White Matter	0.0680 (-0.079, 0.215)	0.364	Reference	
Brainstem Volume	0.0366 (-0.078, 0.151)	0.532	Reference	
Cerebrospinal Fluid	-0.0948 (-0.224, 0.035)	0.151	Reference	
Thalamus (L)	-0.0510 (-0.165, 0.063)	0.380	Reference	
Thalamus (R)	-0.0851 (-0.200, 0.030)	0.146	Reference	

Caudate (L)	0.0395 (-0.083, 0.162)	0.525	Reference	
Caudate (R)	0.0608 (-0.063, 0.185)	0.335	Reference	
Putamen (L)	0.0911 (-0.0367, 0.219)	0.161	Reference	
Putamen (R)	0.1075 (-0.022, 0.237)	0.103	Reference	
Pallidum (L)	0.0488 (-0.079, 0.176)	0.453	Reference	
Pallidum (R)	0.1275 (0.003, 0.252)	0.071	Reference	
Hippocampus (L)	-0.0393 (-0.165, 0.086)	0.659	Reference	
Hippocampus (R)	-0.0767 (-0.202, 0.049)	0.324	Reference	
Amygdala (L)	-0.0604 (-0.185, 0.064)	0.417	Reference	
Amygdala (R)	-0.0666 (-0.187, 0.054)	0.332	Reference	
Accumbens (L)	-0.0261 (-0.154, 0.102)	0.841	Reference	
Accumbens (R)	-0.1326 (-0.264, -0.001)	0.107	Reference	
Deep White Matter Hyperintensities	0.0941 (-0.113, 0.301)	0.372	Reference	
Periventricular White Matter Hyperintensities	0.1525 (0.022, 0.283)	0.022	Reference	

Adjusted for age, sex, BMI, stroke severity, time from initial stroke to MRI scan, head-scanner position, intracranial volume, and head-size scaling factor

Supplementary Table S7. Odds Ratios for Hourly Sleep and Depression.

	**Depression**			
	**Yes**		**No**	
**Sleep (min/day)**	**Odds Ratio**	**p-value**	**Odds Ratio**	**p-value**
0:00-0:59 h	0.989 (0.972-1.005)	0.175	Reference	
1:00-1:59 h	0.972 (0.949-0.995)	0.019	Reference	
2:00-2:59 h	0.952 (0.917-0.988)	0.009	Reference	
3:00-3:59 h	0.947 (0.904-0.991)	0.019	Reference	
4:00-4:59 h	0.958 (0.915-1.004)	0.076	Reference	
5:00-5:59 h	1.006 (0.965-1.050)	0.776	Reference	

Adjusted for age, sex, time from initial stroke to accelerometer study, and season of wear.
